# Empathy and big five personality model in medical students and its relationship to gender and specialty preference: a cross-sectional study

**DOI:** 10.1186/s12909-019-1485-2

**Published:** 2019-02-14

**Authors:** Teresa Guilera, Iolanda Batalla, Carles Forné, Jorge Soler-González

**Affiliations:** 1 Psychiatry Service, Santa Maria University Hospital, Rovira Roure Avenue, 44, 25198 Lleida, Spain; 2 Institute for Biomedical research in Lleida Dr. Pifarré Foundation (IRBLleida), Lleida, Spain; 3 Department of Medicine, Faculty of Medicine, University of Lleida, Lleida, Spain; 4 Department of Basic Medical Sciences, University of Lleida, Lleida, Spain; 5 Biostatistics Unit, Institute for Biomedical Research in Lleida Dr. Pifarré Foundation (IRBLleida), Lleida, Spain

**Keywords:** Big five model, Empathy, Medical education, Personality, Undergraduate

## Abstract

**Background:**

Given the influence that personality can have on empathy, this study explores the relationship between empathy and personality, using three different measures of empathy, and taking into account gender and specialty preference.

**Methods:**

Cross-sectional study. One hundred and ten medical students completed the Jefferson Scale of Physician Empathy, the Interpersonal Reactivity Index, the Empathy Quotient, and the NEO-FFI Big Five personality model**.** Multivariable linear regression was performed to assess the association between personality traits and empathy.

**Results:**

Empathy scales showed weak and moderate correlation with personality. The strongest correlations were observed between IRI-Fantasy and Openness, and between IRI-Personal Distress and Neuroticism. Gender and specialty preference can modify this relationship. The extreme groups of Empathy Quotient had significant differences in most personality traits.

**Conclusions:**

This study confirmed that empathy is related to personality. Using three empathy scales allows personalizing the evaluation of different empathy models and its relation with personality. These results can help to design programs to study if some personalized intervention strategies could improve the empathy in medical students.

**Electronic supplementary material:**

The online version of this article (10.1186/s12909-019-1485-2) contains supplementary material, which is available to authorized users.

## Background

Empathy is the ability to identify and understand the thoughts and feelings of others and to respond with appropriate emotions [1]. It is a complex social emotion difficult to conceptualize and measure [2, 3]. Medical empathy has been defined as the predominantly cognitive attribute that involves the ability to understand patients’ experiences, concerns, and perspectives, and communicate this understanding with the intention of helping [4, 5]. There are different empathy scales based on different theoretical models. The JSPE (Jefferson Scale of Physician Empathy) measures empathy in the context of medical education and patient care [4]. The IRI (Interpersonal Reactivity Index) is a multidimensional approach that permits discrimination between cognitive [IRI-PT (IRI-Perspective Taking) and IRI-FS (IRI-Fantasy Scale)] and affective empathy [IRI-Empathetic Concern (IRI-EC) and IRI-PD (IRI-Personal Distress)] [6, 7]. The EQ (Empathic Quotient) measures cognitive and affective empathy in adults, and is based on the two-dimensional psychological model: empathy and systematization proposed by Baron-Cohen [1, 8]. There is conflicting evidence about the relationship between empathy in medical students with gender and specialty preference. To date higher empathy scores have been observed in women in JSPE [4, 7, 9–18], in IRI-FS and IRI-PD [7], and in EQ [1]. Although other studies report no gender differences [7, 11]. Similarly, some studies of specialty preference show higher empathy scores in medical students with people-oriented versus technology-oriented specialties preference [2, 10, 11, 13, 16, 19], while others do not find a significant difference [11, 14, 17, 20].

Furthermore, personality could be an important variable that could modulate empathy [7, 21]. Personality is defined as the pattern of thoughts, feelings, attitudes, habits and behaviour of each individual that persists over time in different situations distinguishing one individual from others. The NEO Five-Factor Inventory (NEO-FFI), based on the Big Five personality model [22], allows the evaluation of five main factors: Openness to experience, Conscientiousness, Extraversion, Agreeableness and Neuroticism. Personality is normally considered relatively stable. Personality traits might influence empathetic behaviour towards patients, and might play a role in the selection of students for medicine, or in advice concerning suitability of specialty. The associations between empathy and Big Five personality traits in medical education are still underrepresented in the existing literature [23]. Two studies, which used the JSPE among Portuguese medical students [14, 24] concluded that empathy is positively associated with Agreeableness and Openness. Another study, which used IRI in Chinese medical students, found a strong association between Empathic Concern and Agreeableness, and Personal Distress with Neuroticism [23]. We have not found any study using the EQ in medical students. One study carried out with Psychology University students concluded that Agreeableness, Openness, Conscientiousness and Extraversion could be considered predictors of empathy *as* measured with the IRI [25]. A general population study found an association between IRI-PT and Openness and Agreeableness personality traits [26]. Another study with the general population found strong associations between the EQ with Agreeableness and Extraversion [27], but a study of Japanese university students found no association between the EQ and the NEO-FFI [28]. In a four-country multi-centre study of university students that correlated EQ and IRI, with NEO-FFI, the EQ had the highest correlation with Agreeableness, IRI-FS with Openness, and IRI-PD with Neuroticism [29].

Given the influence that personality can have on empathy, the aim of this research is to explore the relationship between empathy and personality, using three different measures of empathy and taking into account gender and specialty preference.

## Methods

This observational cross-sectional study was conducted in a single institution, and the study population consisted of medical students. A description of the project, together with an invitation to participate and a link to access the online questionnaire were send by email from the Faculty of Medicine in Spain. The survey was administered in the 2016/2017 academic year. A total of 669 medical students (70.55% female) were enrolled during the 2016/2017 academic year. 110 medical students completed the survey. Participation was voluntarily with informed consent, and no incentives were offered. The response rate was the 16.44%.

The study was approved by the ethics committee for clinical research (CEIC). The confidentiality was ensured. Only the principal investigator has an access to survey results. The data collected were used exclusively for the purpose of the study.

The online questionnaire included sociodemographic questions (age, gender and academic course) and the following self-report measurement instruments:

1. The JSPE consists of 20 items with scores ranging between 20 and 140. It measures empathy in the context of medical education [4]. The JSPE has been used in most of the studies carried out with medical students. In this study the adapted and validated Spanish version by Alcorta-Garza et al. [30] was used.

2. The IRI is formed by four subscales of seven items each with scores ranging from 0 to 28. Two subscales measure cognitive empathy (IRI-PT and IRI-FS) and two subscales measure affective empathy (IRI-EC and IRI-PD) [6]. IRI-PT measures the spontaneous ability to adopt the perspective of others in real situations of daily life. IRI-FS measures the imaginative ability to put oneself in fictitious situations. IRI-EC measures compassion and concern feelings towards discomfort of others. IRI-PD measures anxiety and discomfort feelings in oneself when observing the negative experiences of others. In the present study, the Spanish validated version of Carrasco-Ortiz et al. [31] has been used.

3. The EQ consists of 60 items, 40 measure empathy and 20 measure control. Scores range from 0 to 80. It measures cognitive and affective empathy in adults. EQ allows classification into four categories, which facilitates the comparison between groups. The cut off for each level is: from 0 to 32 scores is low empathy (average scores in Asperger Syndrome is 20), from 33 to 52 scores: average empathy (average in men 42, average in women 47), from 53 to 63 scores: above average, from 64 to 80 scores high empathy. The version used, which has not been validated in Spanish, was obtained from http://espectroautista.info/EQ-es.html. We have the author’s consent to use the EQ questionnaire [1].

5. Specialty preference classification is based on Hojat’s study [4, 9] which defines two categories of professional preference: people-oriented specialties (Internal Medicine, Family Medicine, Pediatrics, Neurology, Rehabilitation, Psychiatry, Emergency Medicine, Obstetrics and Gynecology, Ophthalmology, Dermatology), and technology-oriented specialties (Surgery and Surgical Specialties, Radiology, Radiation Oncology, Pathology, Anesthesiology).

6. The NEO Five-Factor Inventory (NEO-FFI) is the short form version of one of the most prestigious instruments for the evaluation of normal personality [22]. The NEO-FFI consists of 60 items and evaluates five main factors**.** Openness to experience (O), describes the trend to seek new personal experiences and to creatively conceive the future; Conscientiousness (C) describes responsibility, ability to focus on goals, and discipline to carry them out; Extraversion (E) describes the trend to be open to others in social contexts; Agreeableness (A) describes kindness, respect and tolerance towards others; and Neuroticism (N) describes emotional stability and how to deal with the problems of life. As well as the direct scores, the corresponding percentiles have been obtained based on scales from the Spanish population [32].

### Statistical methods

Quantitative variables were described as means and standard deviations if they were normally distributed. For non-normally distributed quantitative variables, evaluated by the Shapiro-Wilks test, we used medians and interquartile ranges. Frequencies and percentages were used to describe the qualitative variables. Differences between the empathy scales by gender and specialty preference were analysed using the Student’s t-test or the Mann-Whitney U test. Correlations between the empathy and personality variables were assessed by calculating the Spearman’s rho. Multivariable linear regression analysis was performed to assess the association between personality traits (NEO-FFI dimensions) and empathy. We fitted different models for each empathy scale (JSPE, IRI-PT, IRI-FS, IRI-EC, IRI-PD and EQ). The explanatory variables were the NEO-FFI dimensions, adjusting by gender and specialty preference. The selection of variables was performed by backward stepwise regression, removing variables from the model by means of the F test. We explored all first order interactions between adjusting variables (gender and specialty preference) and the personality variables included in the model. The final models were assessed for residual validation. Goodness-of-fit was assessed by means of R-squared and adjusted R-squared.

All statistical tests were two-sided at a significance level of 0.05. Statistical analysis was carried out with the R software. [33].

## Results

Descriptive results of the study sample are shown in Table 1. Most of the medical students were women, from advanced courses, and had a people-oriented specialty preference. The percentile scores for Agreeableness recorded by our students were below those recorded for the Spanish population [32], while those for Openness to experience were higher. Women recorded a higher IRI-EC score (*p* = 0.026). Students with people-oriented specialty preference recorded higher levels of the JSPE (*p* = 0.009) and the IRI-PD (*p* = 0.006) (Additional file 1: Table S1).

**Table 1:** Descriptive analysis of the study sample

Characteristic		*N* = 110
Age (years)		22.0 (20.0–23.0)
Gender (women)		84 (76.4%)
Academic year		
(1st-2nd-3rd)		41 (37.3%)
(4th–5th-6th)		69 (62.7%)
Specialty preference (people-oriented)		80 (72.7%)
Empathy	JSPE	120.5 (111.2–129.8)
	IRI-PT (Perspective Taking)	18.3 (3.9)
	IRI-FS (Fantasy Scale)	18.0 (14.0–23.8)
	IRI-EC (Empathic Concern)	23.0 (20.0–24.0)
	IRI-PD (Personal Distress)	8.0 (5.0–11.0)
	EQ (quantitative scale)	49.0 (41.2–57.0)
	EQ (qualitative scale)	
	Low (0–32)	5 (4.5%)
	Average (33–52)	61 (55.5%)
	Above average (53–63)	36 (32.7%)
	High (64–80)	8 (7.3%)
Personality (NEO-FFI)	Openness to experience (O)	32.1 (6.9)
	Conscientiousness (C)	31.6 (7.9)
	Extraversion (E)	31.7 (7.0)
	Agreeableness (A)	30.8 (7.1)
	Neuroticism (N)	23.0 (8.6)
	Percentile O	75.0 (60.0–95.0)
	Percentile C	40.0 (20.0–70.0)
	Percentile E	55.0 (40.0–85.0)
	Percentile A	25.0 (11.2–53.8)
	Percentile N	55.0 (25.0–75.0)

Quantitative variables are presented as median and interquartile range, or mean and standard deviation; qualitative variables are presented as number and percentage

*JSPE* Jefferson Scale of Physician Empathy, *IRI* Interpersonal Reactivity Index, *EQ* Empathy Quotient, *NEO-FFI* NEO Five-Factor Inventory

The empathy scales showed weak and moderate correlation with all the personality traits (Table 2). The strongest correlations were observed between IRI-FS and Openness, Spearman’s rho (95% confidence interval) of 0.465 (0.305, 0.600); and between IRI-PD and Neuroticism, 0.438 (0.273, 0.578). Medical students with below average EQ scores recorded low scores for Openness to experience, Conscientiousness, Extraversion and Agreeableness, and high scores for Neuroticism. When comparing the extreme groups (low EQ vs high EQ), significant differences were observed for almost all personality traits (Additional file 1: Table S2).

**Table 2:** Correlations between empathy and personality variables

	O	C	E	A	N
JSPE	0.337 (0.160, 0.493) < 0.001	0.088 (− 0.101, 0.270) 0.363	0.249 (0.064, 0.416) 0.009	0.357 (0.182, 0.510) < 0.001	− 0.086 (− 0.269, 0.103) 0.372
IRI-PT	0.299 (0.119, 0.461) 0.001	0.179 (− 0.009, 0.354) 0.062	0.005 (− 0.183, 0.192) 0.960	0.220 (0.034, 0.391) 0.021	− 0.130 (− 0.310, 0.058) 0.174
IRI-FS	0.465 (0.305, 0.600) < 0.001	− 0.157 (− 0.335, 0.031) 0.101	0.208 (0.021, 0.380) 0.029	0.047 (− 0.142, 0.232) 0.629	0.135 (− 0.054, 0.314) 0.161
IRI-EC	0.224 (0.039, 0.395) 0.019	0.049 (− 0.140, 0.234) 0.614	0.288 (0.106, 0.451) 0.002	0.307 (0.127, 0.467) 0.001	0.172 (− 0.016, 0.348) 0.072
IRI-PD	−0.009 (− 0.195, 0.179) 0.929	−0.103 (− 0.285, 0.086) 0.285	−0.224 (− 0.395, − 0.038) 0.019	−0.100 (− 0.282, 0.089) 0.298	0.438 (0.273, 0.578) < 0.001
EQ	0.344 (0.167, 0.499) < 0.001	0.238 (0.053, 0.407) 0.012	0.373 (0.200, 0.524) < 0.001	0.383 (0.210, 0.532) < 0.001	−0.114 (− 0.295, 0.075) 0.235

Spearman’s rho (95% confidence interval), *p*-value

*O* Openness to experience, *C* Conscientiousness, *E* Extraversion, *A* Agreeableness, N Neuroticism, *JSPE* Jefferson Scale of Physician Empathy, *IRI* Interpersonal Reactivity Index, *PT* Perspective Taking, *FS* Fantasy Scale, *EC* Empathic Concern, *PD* Personal Distress, *EQ* Empathy Quotient

**Fig. 1:**
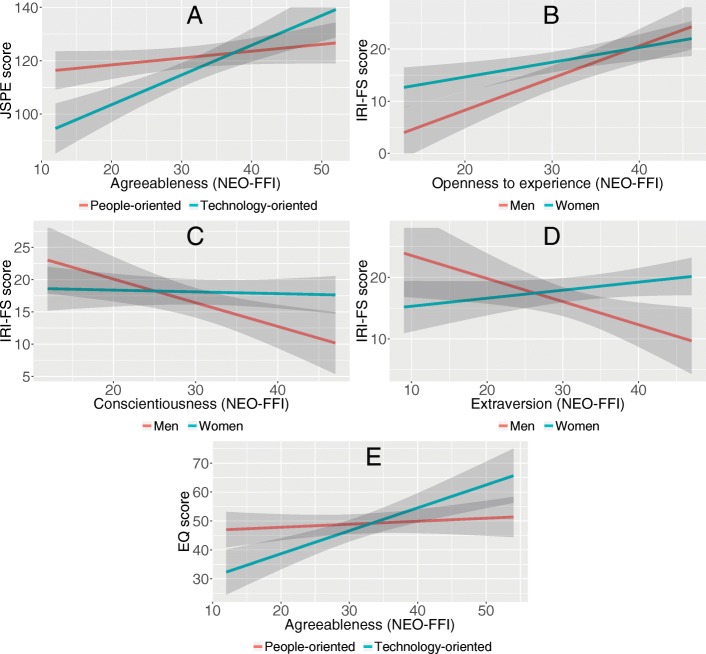
Interactions of gender or specialty preference with personality traits from multivariable models for empathy scales. **a**: Model for JSPE score: interaction between Agreeableness and specialty preference; **b**: Model for IRI-FS score: interaction between Openness to experience and gender; **c**: Model for IRI-FS score: interaction between Conscientiousness and gender; **d**: Model for IRI-FS score: interaction between Extraversion and gender; **e**: Model for EQ score: interaction between Agreeableness and specialty preference. JSPE, Jefferson Scale of Physician Empathy; IRI, Interpersonal Reactivity Index; FS, Fantasy Scale; EQ, Empathy Quotient

Table 3 shows results of multivariable analysis for each empathy scale. In addition to specialty preference, the JSPE was associated significantly with Openness personality trait, while a higher JSPE score was associated with higher Agreeableness only in students with technology-oriented specialty preference (Fig. 1a). IRI-PT was significantly associated with all dimensions of NEO-FFI. Higher IRI-PT scores were associated with higher Agreeableness, Openness and Conscientiousness, and low Extraversion and Neuroticism scores. Regardless of gender and specialty preference, IRI-FS was associated with Openness personality trait, with higher effect among men (Fig. 1b). Higher IRI-FS was associated with lower Conscientiousness (Fig. 1c) and lower Extraversion only among men (Fig. 1d). IRI-EC was significantly associated with Extraversion, Agreeableness and Neuroticism scores. IRI-PD was only associated with Neuroticism, furthermore to specialty preference with lower IRI-PD scores for students with technology-oriented specialty preference. The EQ score was significantly associated with Openness, Conscientiousness and Extraversion, while a higher EQ score was associated with higher Agreeableness score in students with technology-oriented specialty preference (Fig. 1e). Big five personality traits, taken into account gender and specialty preference, explained 24–43% of variance of the empathy scores.

**Table 3:** Multivariable linear regression analysis between empathy and personality, adjusted by gender and specialty preference

		Beta	SE	*p*-value	Multiple R^2^
JSPE	Constant	98.50	6.42	< 0.001	0.366
	Men	−0.24	2.18	0.914	
	Technology-oriented	−32.05	8.53	< 0.001	
	O	0.47	0.14	0.001	
	A	0.26	0.16	0.110	
	Technology-oriented*A	0.86	0.27	0.002	
IRI-PT	Constant	9.57	3.01	0.002	0.287
	Men	−0.50	0.81	0.542	
	Technology-oriented	0.02	0.75	0.977	
	O	0.24	0.05	< 0.001	
	C	0.12	0.04	0.005	
	E	−0.12	0.06	0.031	
	A	0.12	0.05	0.015	
	N	−0.12	0.04	0.009	
IRI-FS	Constant	6.49	4.07	0.114	0.372
	Men	13.56	8.23	0.102	
	Technology-oriented	−0.70	1.08	0.520	
	O	0.28	0.09	0.003	
	C	−0.03	0.07	0.707	
	E	0.13	0.08	0.116	
	Men*O	0.33	0.15	0.033	
	Men*C	−0.34	0.15	0.024	
	Men*E	−0.50	0.18	0.005	
IRI-EC	Constant	8.99	2.76	0.002	0.242
	Men	−1.41	0.86	0.106	
	Technology-oriented	0.13	0.81	0.870	
	E	0.12	0.06	0.046	
	A	0.23	0.05	< 0.001	
	N	0.10	0.05	0.038	
IRI-PD	Constant	1.90	1.18	0.111	0.326
	Men	0.54	0.92	0.563	
	Technology-oriented	−2.17	0.87	0.014	
	N	0.30	0.05	< 0.001	
EQ	Constant	11.96	6.45	0.067	0.431
	Men	−0.63	1.83	0.733	
	Technology-oriented	−22.93	7.07	0.002	
	O	0.43	0.12	< 0.001	
	C	0.34	0.10	0.001	
	E	0.31	0.12	0.010	
	A	0.10	0.14	0.448	
	Technology-oriented*A	0.69	0.23	0.003	

*SE* standard error, *O* Openness to experience, *C* Conscientiousness, *E* Extraversion, *A*, Agreeableness, *N* Neuroticism, *JSPE* Jefferson Scale of Physician Empathy, *IRI* Interpersonal Reactivity Index, *PT* Perspective Taking, *FS* Fantasy Scale, *EC* Empathic Concern, *PD* Personal Distress, *EQ* Empathy Quotient

* stands for interaction term

## Discussion

This study explores the relationship between three empathy scales and the Big Five personality traits in medical students, taking into account gender and specialty preference, not yet been explored in previous studies.

In our sample, there were no gender differences in the JSPE medical empathy scale as in other studies [11] nor in the EQ. However, higher IRI-EC values were observed among women, as already Neumann et al. [7] observed. Female superiority in this ability according to the evolutionary theory of gender might explain these results [34].

In medical students with people-oriented specialty preference, a higher score was observed in the JSPE and IRI-PD, as found in previous studies [4, 9, 11, 13, 16, 19]. The JSPE specifically evaluates empathy in the medical context, the focus of attention being the patient. In some technology oriented specialties empathy maybe is less apparent or important in the doctor-patient relationship [4, 9]. IRI-PD evaluates anxiety and discomfort feelings when observing the patients’ suffering and discomfort. Therefore, learning to manage anxiety could improve social relationships and empathic behaviour [35].

Regarding the Big Five personality model, the Openness to experience percentile of medical students was higher and the Agreeableness lower than those in the Spanish population. Neuroticism, Extraversion and Conscientiousness percentiles were similar [32]. Although Agreeableness implies tolerance and respect for others, lower Agreeableness score reflects more scepticism and competitiveness, which may be requisite traits in science.

In our sample, JSPE was positively associated with Openness, and with Agreeableness only in students with technology-oriented specialty preference. These results are similar to previous studies in Portuguese medical students [14, 24].

IRI-PT was positively associated with Agreeableness, Openness and Conscientiousness, and negatively with Extraversion and Neuroticism. The recent study in Chinese medical students show similar results except Extraversion [23].

IRI-FS was positively associated with Openness, and negatively with Conscientiousness and Extraversion, similar to Melchers’ results [29]. We observed a higher effect among men in these associations. In our opinion, probably an open and more flexible attitude fosters fantasy, imagination and creativity.

IRI-EC was positively associated with Extraversion, Agreeableness and Neuroticism. Song & Shi [23] report similar results except in Extraversion. Cultural differences in Chinese population could explain mixed results in Extraversion [36].

IRI-PD was only positively associated with Neuroticism, as found in other studies [23, 29]. Technology-oriented specialty preference was associated with a decrease of IRI-PD. The relationship with anxiety and the possibility of improving it could be one of the main goals of personalized interventions [37].

Song’s study does not use IRI-FS, as some authors considered subscales IRI-PT and IRI-EC relevant for patient care, underestimating IRI-FS and IRI-PD [19, 38, 39]. We think that neither IRI-FS nor IRI-PD can be dismissed, since they provide relevant information as to how the student approaches the doctor-patient relationship [35].

We have not found previous studies, to compare the results, with the EQ in medical students. Medical students with below average EQ scores recorded low scores for Openness. However, we found EQ was positively associated with Openness, Conscientiousness and Extraversion, and associated with Agreeableness in students with technology-oriented specialty preference. These results partial match with those of Melchers’ and collaborators [29]. We highlight that some students who prefer more technological specialties are those who had the highest Agreeableness scores. Students with extreme empathy scores (low and high) showed a differential personality pattern; those with high empathy sometimes could avoid people-oriented specialties to not get emotionally involved and not increase the basal anxiety. Those with low empathy were more introverted, anxious and they had a less open attitude.

Gender and specialty preference could modify the relationship between empathy and personality. Thus gender and specialty preference are variables that should be taken into account in empathy research in medical students. The explanatory and predictive capacity of this relationship is limited, supporting the consideration of empathy as a complex multidimensional socio-emotional competency [40, 41].

The results obtained with the three empathy scales can help to decide the empathy model to be used to develop personalized empathy interventions. The JSPE would be specific to evaluate medical empathy. Multidimensional model of the IRI allows study of both affective and cognitive empathy, and the EQ allows identifying extreme empathy scores. Probably, personality could be a good predictor in vulnerable medical students with extreme scores of empathy who benefit most from a specialty choice advice [35].

Given the differentiation between affective and cognitive empathy and that some dimensions of empathy can, perhaps, be taught and modified, these results *may* help to design programs to study *the effects of* personalized intervention strategies [23]. The results of two pilot studies recently published by our team show the wide acceptance of two psychoeducational intervention (sensory deprivation and shadowing patients) easily implemented in undergraduate medical studies [42, 43]. Accordingly, we proposed to improve cognitive empathy by increasing attitudes related to the agreeableness trait. People with low agreeableness may have difficulty focusing their attention on others, hence, we propose to train perspective taking in a fictional context with intervention strategies that will help them to direct the focus of attention towards the other patients, without fear or defensive behaviours that will distance them from patients. We also propose to improve affective empathy by modulating neuroticism and anxiety related to empathic concern and personal distress, especially in students with people-oriented specialty preference, who tend to have higher anxiety in their relationship with patients. According to our results, we might teach medical students to accurately perceive and identify their emotions and those of others. Although they could improve the scores of Agreeableness and decrease Neuroticism scores, above all it would improve empathic behaviour and patient care.

This study has different limitations. This observational cross-sectional study was conducted in a single institution. Although the sample of 110 medical students may not be representative of the general medical student population, it opens empathy research lines to be evaluated in future multi-centre studies. Our students were participating in another study at the same time, and their collaboration was not always easy.

Our study provides new perspectives in psychoeducational interventions to advise and improve empathy in medical students with extreme values. Although we have experience in both individualized and group interventions, we are aware of the need for future methodologically better-supported studies to verify or confirm that personalized intervention strategies could improve empathy in medical students.

We used self-report instruments that inform us of the perception that the individual has of himself and of his abilities. Our team is interested in simplifying and optimizing the correlation process of psychometric instruments traditionally used, and new biometric devices that provide more objective information about emotions [44].

The low proportion of males was a limitation of our study but reflects the increased proportion of women attending medical school; 70% in Spain [45], and is similar to gender in our Faculty.

In the Big Five model, NEO-FFI-R structure analyses show only a slight improvement in Openness and Agreeableness reliability, but correlations between the five dimensions of the NEO-FFI and the NEO-FFI-R are similar to each other, and they correspond to those expected. The choice of one or the other depends on the necessity to compare results with other studies [46]. For this reason, in this study we chose the NEO-FFI to be able to compare our results with the version used in previous studies of personality in medical students [14, 23, 24].

## Conclusions

This study confirmed that the empathy is related to personality. Gender and specialty preference can modify this relationship. Using three different measures of empathy allows personalizing the evaluation of the different empathy models of empathy and its relation with personality traits. The strongest correlations were observed between IRI-Fantasy Scale and Openness to experience, and between IRI-Personal Distress and Neuroticism. These results can help to design programs to study whether some personalized intervention strategies could improve the empathy in medical students by increasing perspective taking ability and decreasing anxiety. However, more studies are needed to verify these hypotheses. Although they could improve the scores of Agreeableness and decrease Neuroticism scores, above all it would improve empathic behaviour and patient care.

## Additional file

Additional file 1: **Table S1**. Differences between empathy scales by gender and specialty preference. Variables are described with medians and interquartile ranges, except for IRI-PT that is described with mean and standard deviation. JSPE, Jefferson Scale of Physician Empathy; IRI, Interpersonal Reactivity Index; PT, Perspective Taking; FS, Fantasy Scale; EC, Empathic Concern; PD, Personal Distress; EQ, Empathy Quotient. a. Mann-Whitney U test, except for IRI-TP that is analysed with the t test. **Table S2**. Bivariate analysis between Empathy Quotient (qualitative scale) and personality. Variables are presented as median and interquartile range, or mean and standard deviation. *P*-values correspond to the Mann-Whitney U test or the t-test comparing the extreme groups: low Empathy Quotient score vs high Empathy Quotient score. (DOCX 16 kb)

## Abbreviations

A: Agreeableness
C: Conscientiousness
E: Extraversion
EQ: Empathy Quotient
IRI: Interpersonal Reactivity Index
IRI-EC: Interpersonal Reactivity Index-Subscale Empathetic Concern
IRI-FS: Interpersonal Reactivity Index- Subscale Fantasy Scale
IRI-PD: Interpersonal Reactivity Index- Subscale Personal Distress
IRI-PT: Interpersonal Reactivity Index-Subscale Perspective Taking
JSPE: Jefferson Scale of Physician Empathy
N: Neuroticism
NEO-FFI: NEO Five-Factor Inventory
O: Openness to experience
