# Supplementary material for: Empathy and big five personality model in medical students and its relationship to gender and specialty preference: a cross-sectional study
Source: BMC Med Educ. 2019 Feb 14;19:57. doi: 10.1186/s12909-019-1485-2 (PMC6376790; doi:10.1186/s12909-019-1485-2)
Supplement: Supplementary file 1 — Table S1. Differences between empathy scales by gender and specialty preference. Variables are described with medians and interquartile ranges, except for IRI-PT that is described with mean and standard deviation. JSPE, Jefferson Scale of Physician Empathy; IRI, Interpersonal Reactivity Index; PT, Perspective Taking; FS, Fantasy Scale; EC, Empathic Concern; PD, Personal Distress; EQ, Empathy Quotient. a. Mann-Whitney U test, except for IRI-TP that is analysed with the t test. Table S2. Bivariate analysis between Empathy Quotient (qualitative scale) and personality. Variables are presented as median and interquartile range, or mean and standard deviation. P-values correspond to the Mann-Whitney U test or the t-test comparing the extreme groups: low Empathy Quotient score vs high Empathy Quotient score. (DOCX 16 kb) [file 12909_2019_1485_MOESM1_ESM.docx]

**Additional files.**

Additional file 1: **Table S1** Differences between empathy scales by gender and specialty preference.

|  | **Gender** | |  | **Specialty preference** | |  |
| --- | --- | --- | --- | --- | --- | --- |
|  | **Men**  **n=26** | **Women**  **n=84** | **p-value^a^** | **People-oriented**  **n=80** | **Technology-oriented**  **n=30** | **p-value^a^** |
| **JSPE** | 120.5 (112.0 - 127.5) | 120.5 (110.8 – 130.0) | 0.657 | 122.5 (113.8 - 130.0) | 113.0 (101.5 - 127.5) | 0.009 |
| ***IRI-PT*** | 17.9 (4.0) | 18.4 (3.9) | 0.573 | 18.4 (3.5) | 17.9 (5.0) | 0.606 |
| ***IRI-FS*** | 17.0 (11.2 - 22.0) | 18.0 (15.0 - 24.0) | 0.203 | 18.0 (15.0 - 24.0) | 16.0 (12.2 - 20.0) | 0.054 |
| ***IRI-EC*** | 20.0 (18.2 - 23.8) | 23.0 (21.0 - 24.2) | 0.026 | 22.5 (20.0 - 24.0) | 23.0 (20.0 - 24.8) | 0.589 |
| ***IRI-PD*** | 6.5 (4.2 - 9.8) | 8.0 (5.0 - 11.2) | 0.387 | 9.0 (5.0 - 12.0) | 6.0 (3.0 - 7.8) | 0.006 |
| **EQ** | 46.5 (41.0 - 55.5) | 50.0 (43.0 - 57.2) | 0.402 | 50.0 (43.8 - 56.2) | 45.0 (37.0 - 58.5) | 0.278 |
| Variables are described with medians and interquartile ranges, except for IRI-PT that is described with mean and standard deviation.  JSPE, Jefferson Scale of Physician Empathy; IRI, Interpersonal Reactivity Index; PT, Perspective Taking; FS, Fantasy Scale; EC, Empathic Concern; PD, Personal Distress; EQ, Empathy Quotient.  a. Mann-Whitney U test, except for IRI-TP that is analysed with the t test. | | | | | | |

**Table S2** Bivariate analysis between Empathy Quotient (qualitative scale) and personality.

|  | **Empathy Quotient (qualitative scale)** | | | |  |
| --- | --- | --- | --- | --- | --- |
|  | **Low**  **n=5** | **Average**  **n=61** | **Above average**  **n=36** | **High**  **n=8** | **p-value**  **(Low vs High)** |
| Openness to experience (O) | 26.6 (6.7) | 30.3 (6.6) | 34.9 (6.1) | 36.1 (6.5) | 0.035 |
| Conscientiousness (C) | 22.6 (8.0) | 31.2 (7.6) | 33.0 (7.6) | 34.1 (9.0) | 0.039 |
| Extraversion (E) | 22.6 (8.4) | 30.7 (6.2) | 33.7 (7.1) | 36.6 (5.4) | 0.015 |
| Agreeableness (A) | 24.2 (9.7) | 29.0 (6.9) | 34.5 (5.5) | 31.9 (6.6) | 0.168 |
| Neuroticism (N) | 27.2 (10.4) | 23.5 (8.8) | 21.7 (8.6) | 22.8 (6.1) | 0.422 |
| Percentile O | 65.0 (45.0 - 65.0) | 70.0 (40.0 - 85.0) | 80.0 (63.8 - 95.2) | 88.0 (72.5 - 97.5) | 0.032 |
| Percentile C | 15.0 (3.0 - 30.0) | 35.0 (15.0 - 60.0) | 52.5 (32.5 - 80.0) | 50.0 (23.8 - 91.2) | 0.067 |
| Percentile E | 25.0 (20.0 - 30.0) | 50.0 (40.0 - 75.0) | 75.0 (48.8 - 95.0) | 85.0 (73.8 - 92.0) | 0.008 |
| Percentile A | 5.0 (1.0 - 10.0) | 20.0 (10.0 - 45.0) | 45.0 (30.0 - 61.2) | 27.5 (13.8 - 52.5) | 0.077 |
| Percentile N | 70.0 (65.0 - 80.0) | 55.0 (30.0 - 75.0) | 55.0 (15.0 - 70.0) | 55.0 (43.8 - 61.2) | 0.300 |
| Variables are presented as median and interquartile range, or mean and standard deviation. P-values correspond to the Mann-Whitney U test or the t-test comparing the extreme groups: low Empathy Quotient score vs high Empathy Quotient score. | | | | | |
